# Characterization of Fatty Acids as Biobased Organic Materials for Latent Heat Storage

**DOI:** 10.3390/ma14164707

**Published:** 2021-08-20

**Authors:** Marie Duquesne, Clément Mailhé, Stefania Doppiu, Jean-Luc Dauvergne, Sergio Santos-Moreno, Alexandre Godin, Guillaume Fleury, Fabien Rouault, Elena Palomo del Barrio

**Affiliations:** 1 Univ. Bordeaux, CNRS, I2M Bordeaux, Bâtiment A11, 351 cours de la Libération, CEDEX, 33405 Talence, France; clemailhe@gmail.com; 2 Centre for Cooperative Research on Alternative Energies (CICenergiGUNE), Basque Research and Technology Alliance (BRTA), Alava Technology Park, 01510 Vitoria-Gasteiz, Spain; sdoppiu@cicenergigune.com (S.D.); jldauvergne@cicenergigune.com (J.-L.D.); ssantos@cicenergigune.com (S.S.-M.); epalomo@cicenergigune.com (E.P.d.B.); 3 TECNALIA, Basque Research and Technology Alliance (BRTA), Parque Tecnológico de San Sebastián, 20009 Donostia-San Sebastián, Spain; 4 Applied Physics II, University of the Basque Country UPV-EHU, 48940 Leioa, Spain; 5 Amplitude, 11 Avenue de Canteranne, Cité de la Photonique, Bâtiment MEROPA, 33600 Pessac, France; alexandre.godin@amplitude-laser.com; 6 Univ. Bordeaux, CNRS, Bordeaux INP, LCPO—UMR5629, 16 Avenue Pey Berland, CEDEX, 33607 Pessac, France; guillaume.fleury@u-bordeaux.fr; 7 Escuela de Construcción, Facultad de Ingeniería, Pontificia Universidad Católica de Chile, Av. Libertador Bernardo O’Higgins 340, Santiago 8331150, Chile; frouault@uc.cl; 8 Ikerbasque, Basque Foundation for Science, 48013 Bilbao, Spain

**Keywords:** thermal energy storage, biobased phase change materials, fatty acids, thermophysical characterization

## Abstract

This work aims to characterize phase change materials (PCM) for thermal energy storage in buildings (thermal comfort). Fatty acids, biobased organic PCM, are attractive candidates for integration into active or passive storage systems for targeted application. Three pure fatty acids (capric, myristic and palmitic acids) and two eutectic mixtures (capric-myristic and capric-palmitic acids) are studied in this paper. Although the main storage properties of pure fatty acids have already been investigated and reported in the literature, the information available on the eutectic mixtures is very limited (only melting temperature and enthalpy). This paper presents a complete experimental characterization of these pure and mixed fatty acids, including measurements of their main thermophysical properties (melting temperature and enthalpy, specific heats and densities in solid and liquid states, thermal conductivity, thermal diffusivity as well as viscosity) and the properties of interest regarding the system integrating the PCM (energy density, volume expansion). The storage performances of the studied mixtures are also compared to those of most commonly used PCM (salt hydrates and paraffins).

## 1. Introduction

Latent heat thermal energy storage (LHTES) systems are a viable solution for several applications such as building, food industry, electronics, and transport, due to their ability to store a large amount of heat in quasi-isothermal conditions. The main criteria when designing or selecting a thermal energy storage (TES) system are energy efficiency, liability and cost-effectiveness [1]. This work is focused on LHTES systems in which the charge period corresponds to the melting of the phase change material (PCM) and the discharge to its crystallization. The charge/discharge cycles and the associated performances are strongly influenced by the nature of the PCM and their properties (melting temperature and enthalpy, thermal conductivity, etc.) [2,3,4].

The Interreg SUDOE SUDOKET project aims for sustainable urban development, with a final objective to contribute to improving the energy efficiency of existing buildings. In this framework, our work is focused on the thermal regulation of the indoor environment of buildings using a LHTES system as an alternative to conventional heating and air-conditioning systems. A free cooling device based on a LHTES system can manage building overheating, storing the excess heat in the melting process of the PCM during daytime [5]. The melting temperature of the PCM should be close to the maximum limit temperature (around 25 °C). Then, the stored heat can be released to the outdoor environment when the air temperature is below the melting temperature. Once the PCM is completely solidified, the LHTES system is ready for a new cycle. Most previously developed LHTES systems [5,6,7] are based on paraffin waxes.

Since the 1970s, many PCM have been studied for TES applications in buildings and in solar systems [1,2,3,4,8,9,10,11,12,13]. The use of PCM for TES systems in the 20–25 °C (293–298 K) range is being increasingly studied as it corresponds to ambient temperature applications which are of widespread concern, especially regarding thermal comfort in buildings or vehicles [14,15,16]. The use of PCM-based TES systems succeeds in maintaining suitable temperature conditions and is also sustainable and affordable [16,17]. Different classes of PCM have been studied over the years to fit TES applications. The first PCM class was hydrated salts because of their low costs (<1 €·kg^−1^), high energy densities (60–180 kWh·m^−3^), melting temperature ranging from −30 °C to 120 °C and relatively high thermal conductivities (0.4–0.8 W^−1^·m^−1^·K^−1^) [12,18,19,20]. However, they have some drawbacks such as significant undercooling degrees and stability issues due to incongruent melting and corrosiveness [10,13,21,22]. To overcome such issues, research has been focused on organic PCM and their mixtures (paraffin waxes, fatty acids and sugar alcohols [23], etc.).

The organic PCM commonly investigated for LHTES are paraffin waxes, fatty acids and their derivatives (fatty alcohols, fatty esters, and triglycerides), diols and polyethylene glycol (PEG). In a previous study [22], a screening of fatty acids and fatty acid mixtures was performed in order to identify suitable candidates for integration as PCM in a building integrated TES system solely based on their melting/solidification temperature range. This work led to the identification of two new eutectic mixtures of potential interest: capric acid + myristic acid (CA/MA) and capric acid + palmitic acid (CA/PA). The three pure fatty acids composing these mixtures are well referenced in the literature. On the other hand, the available information on eutectic mixtures of fatty acids in the literature is limited to their composition, melting temperature, and enthalpy of fusion. Hence, the novelty of this work lies in the complete characterization of the thermophysical properties (melting temperature and enthalpy, specific heats, densities in liquid and solid phases, volume expansion, thermal conductivity, diffusivity, viscosity and energy density) of these two eutectic fatty acid-mixtures: CA/MA and CA/PA. Finally, the storage performances of the studied mixtures are compared to those of the most commonly used PCM (salt hydrates and paraffin waxes).

## 2. Materials and Methods

### 2.1. Materials

#### 2.1.1. Selection of Biobased PCM

Over an extensive review of biobased PCM for the thermal regulation of the indoor environment [22], a pre-screening of fatty acids was made based on technical and practical requirements such as:

- phase change transition in the range of thermal comfort (21–25 °C),
- high density and a high phase change enthalpy for a large energy storage density
- high thermal conductivity and diffusivity to favor heat transfers
- low volume expansion to avoid damaging the system integrating the selected PCM
- fast kinetics of crystallization for a fast release of the stored heat
- low undercooling to decrease the risk of cooling the PCM without solidification
- cost/sustainability/hazard

The use of fatty acids as potential PCM for thermal energy storage began in 1989 with the pioneering work of Feldman et al. [24]. Fatty acids are carboxylic acids (–COOH) with a straight alkyl chain which normally contains an even number of carbon atoms that can be classified into saturated and unsaturated, depending on the absence or presence of double bonds in the alkyl chain, respectively. They are generally widespread in triglycerides composed of three fatty acids esterified to a glycerol backbone, and they can be extracted by hydrolyzing the triglyceride [25]. Conversely to salt hydrates, these PCM melt congruently, ensuring their stability to thermal cycling. Their melting temperatures allow for application at low temperatures (<100 °C). Moreover, they appear to be a promising biobased alternative to petroleum-based PCM for thermal energy storage applications at low temperatures [26,27,28]. Their melting temperatures range from −23 °C to 78 °C [14,29,30,31], with melting enthalpies from 100 and 300 J·g^−1^. In addition, they are non-toxic, non-corrosive, and non-flammable, have low vapor pressure, exhibit low undercooling upon phase change and are low-cost.

Among the five PCM, three pure fatty acids and two eutectic mixtures have been selected for a complete thermophysical characterization: capric acid (CA), myristic acid (MA), palmitic acid (PA) acids, capric/myristic acids mixture (CA/MA) and capric/palmitic acids mixture (CA/PA). Their properties are compared to the properties of commercial paraffins waxes (petroleum derivatives) such as the RT21, RT25, and RT26 from Rubitherm (Rubitherm, Berlin, Germany) [32] or the PureTemp 25 (PureTemp, Minneapolis, MN, USA) [33] in Table 1.

This comparison shows that fatty acids might be available in the required temperature range as an eco-friendly alternative [22]. Although their applicability has been investigated before as PCM, their integration in large-scale latent heat storage systems still needs to be studied. Finally, fatty acids are economically competitive with paraffin waxes considering an average price of 3 €/kg [37] in the international market.

#### 2.1.2. Description of Selected Fatty Acids and Their Eutectic Mixtures

The CAS number and the stoichiometric formula of capric (CA), myristic (MA), and palmitic (PA) acids are presented in Table 2. These three highly pure fatty acids were purchased from different suppliers.

Two eutectics mixtures were also studied capric acid–myristic acid (CA/MA), and capric acid–palmitic acid (CA/PA). The corresponding compositions have already been determined by Sari et al. and Duquesne et al. [22,38,39,40,41]. Table 3 shows the composition of both eutectic mixtures in terms of the molar fraction. The eutectic binary system is prepared with a Mettler Toledo weighing scale (Mettler Toledo, Bordeaux, France), which has an accuracy of 0.03 mg. Batches of 200 mg are prepared by first weighing the first compound in its solidified form (powder) in an aluminum weighing pan. It is then heated up to its melting and cooled down to its solidification. The second compound is added following the same protocol. The whole sample is heated up to its melting point, mixed to homogenize it, then cooled down until its solidification and weighed again. It should be mentioned that the sample preparation is not made in a controlled atmosphere as fatty acids do not show mixing behaviors with water and are not altered by the environment in the time scale of these experiments.

### 2.2. Measurement Methods

This section provides a description of all experimental methods used for the determination of the considered thermophysical properties. The measurement uncertainties are indicated for each property directly measured. The results section will be recalling uncertainties and will also present the uncertainties of derived properties either obtained through error propagation or repeated measurements.

#### 2.2.1. Melting Temperatures and Enthalpies

The pure fatty acids were prepared in powder form, the eutectic mixtures following the aforementioned protocol (§ 2.1.2); 10–15 mg samples were weighed and placed inside open alumina crucibles.

A DSC 131 differential scanning calorimeter from SETARAM (KEP Technologies, Caluire-et-Cuire, France) combined with a nitrogen cooling system (to reach a temperature below ambient) was used to determine the melting temperature and enthalpy of the studied materials. The DSC was calibrated with four calibration standards: gallium, tin, lead (99.999% + pure each), and indium (99.995% pure), ensuring an excellent accuracy in the temperature range from 29 to 330 °C. Consequently, melting temperatures ($T_{m}$) and melting enthalpies ($\Delta H_{m}$) were measured with an uncertainty of ±0.5 °C and 5%, respectively. Prior to the measurements, a blank run is performed for the baseline correction and a first rapid heating/cooling cycle is completed to ensure an adequate sample/crucible contact. Three heating/cooling runs were then performed, two at 1 °C·min^−1^ and the last at 0.3 °C·min^−1^, all from 5 °C (278 K) to 10 °C above the melting temperature (melting temperatures extracted from literature data).

The interpretation of the DSC curves was made on the software SETSOFT 2000 (KEP Technologies, Caluire-et-Cuire, France), according to the guidelines stated in [42]. If a clear baseline can be identified, the melting temperature is chosen as the onset temperature on the DSC curve, otherwise, the peak temperature is considered. If possible, the peak temperature is not selected as transition temperature, as its position is affected by the heating rate (unlike the onset temperature). Therefore, to make sure that the heating had no influence, a comparison is made between peak temperatures obtained at both heating rates. If no deviation is observed between the results, we can assume that the peak temperature is a satisfying depiction of the transition temperature.

#### 2.2.2. Densities

The densities of the five PCM studied were measured for their liquid and solid states. For the liquid, the density of the tested samples was measured by weighing and directly measuring the volume of melted material in a 10 mL graduated test tube with a 0.1mL reading uncertainty using a Mettler Toledo weighing scale with a 0.03 mg accuracy. A mass of 4–6 g of fatty acids powder was introduced into the test tube and placed in a Nabatherm P330 furnace (Nabatherm, Lilienthal, Germany) whose temperature is, first, set slightly above the melting temperature of the tested sample. Once completely melted, the temperature of the furnace is raised by 10 °C (10 K) increments for a total of 5 measurements per sample. At each temperature, an isothermal level is set to ensure the uniformity of temperature in the sample. At the end of each level, a visual reading of the volume of melted material in the graduated tube is made. The furnace temperature is controlled with a ±0.1 °C accuracy and the stability of the sample temperature is controlled via a thermocouple glued to the glass tube. The temperature difference between the furnace temperature and the sample temperature at equilibrium is insignificant (<0.1 °C). A separate experiment was made prior to the measurement to assess the changes of the glass tube volume with temperature and measurements were corrected accordingly if needed.

Regarding the solid state, the density was determined by helium pycnometry using the Accupyc II 1340 pycnometer from Micromeritics (Norcross, GA, USA) at ambient temperature ($T_{amb}$ = 25 °C). This technique uses the gas displacement in a compartment of known volume to calculate the known mass sample volume within it. Stainless steel balls with an established volume (1, 3.5 or 10 cm^3^) were used as references before each measurement (10 cycles of compression/decompression). The accuracy was about ±0.2% of the measured volume. The solid samples were weighed (1–2 g) using a Mettler Toledo scale with a 0.001 mg accuracy, and then were put into the 10 cm^3^ cell and closed with a microporous lid to start the measurement (the introduced mass being known).

#### 2.2.3. Specific Heat

The determination of the specific heat of the studied materials was carried out with a Q2500 calorimeter from TA instruments (New Castle, DE, USA). For this instrument, temperature and enthalpy were calibrated using sapphire and indium standards: the accuracies were estimated at ±1 K and ±0.02 J/g, respectively. Samples of 10 mg weighed using a Mettler Toledo scale with a 0.001 mg accuracy were placed in closed aluminum crucibles and the measurements were made in a controlled atmosphere (Argon, 50 mL·min^−1^). In these experiments, the sapphire method (according to DIN 51007) was applied. Hence, after the sapphire calibration curve, a heating and cooling, continuous ramp was applied to the samples (2 K·min^−1^). The specific heat values were determined in a single scan by using a normalization factor to the heat flow values (direct mode).

Regarding the specific heat of the samples in liquid state, the measurement was carried out only at the melting temperature, as its variation depending on the temperature is sufficiently low to be considered constant for these PCM, according to Kahwaji et al. [28].

The specific heat of the samples in solid state was measured from 5 °C (278 K), by the means of a nitrogen cooling system, up to a few degrees below the melting temperature of each material.

#### 2.2.4. Thermal Conductivities and Diffusivities

The transient Hot Disk method was used to determine thermal conductivity and diffusivities of each solid state as described in [43] and following the norm Hot Disk ISO 22007-2:2015. The samples for this test were cylindrical tablets with a diameter of 40 mm and a thickness of 10 mm. Regarding the preparation of the eutectic samples, the same protocol as for DSC samples was employed. For each sample, a series of 6 experiments of 20 s was performed at ambient temperature (25 °C, 298 K) with a TPS 2500 instrument equipped with a Kapton insulated sensor (Hot Disk, Gothenburg, Sweden) of 2 mm diameter. The power applied to the rear face of the sample was 4 mW.

#### 2.2.5. Viscosity Measurements

The viscosity measurements were carried out using the rheometer Anton Paar MCR 51 equipped with a temperature control module P-PTD 200 (Anton Paar, Graz, Austria), with a temperature range from −5 °C to 200 °C (±1 °C). The gap of the 50 mm cone/plate geometry was set at 0.207 mm. A 500 mg powder sample was spread on the bottom plate and melted down. Afterwards, the upper plate was pushed down to confine the melted fatty acid.

The Newtonian behavior of the liquid state was first established by determining the viscosity profile as a function of the shear rate. For that test, the temperature was set 10 °C above the melting temperature and the viscosity was scanned within a range of shear rates from 10 s^−1^ to 300 s^−1^. Once the Newtonian behavior was confirmed, the viscosity was measured at a constant shear rate of 100 s^−1^, decreasing the temperature from 10–20 °C above the melting temperature by steps of 5 °C until crystallization started. Each 5 °C step was composed of a ramp of 1 °C·min^−1^ and then an isotherm of 2 min. Viscosity was measured at the end of the isothermal component of each step.

## 3. Results

### 3.1. Melting Temperatures and Enthalpies

Table 4 presents the melting temperatures and enthalpies of fatty acids and their eutectic mixtures and allows the comparison of our results to those in the literature.

The melting temperature is roughly correlated to the length of the molecule which determines the strength of the crystal lattice and the energy required to disrupt it. Thus, the melting temperature increases according to the carbon number in the molecule as presented by the results of our measurements (see the 2nd column in Table 4).

The eutectic mixtures CA/MA and CA/PA exhibit lower melting temperatures than those of the pure materials while having interesting melting enthalpies. These eutectic mixtures are rich in CA and relatively poor in MA and PA (<20 mol%). The compound in minority seeks to insert itself into the crystal lattice of the compound in majority (here CA). This could induce an increase in the average distance between CA molecules which tends to reduce the strength of the crystal lattice leading to a lower energy required to disrupt it.

**Figure 1:**
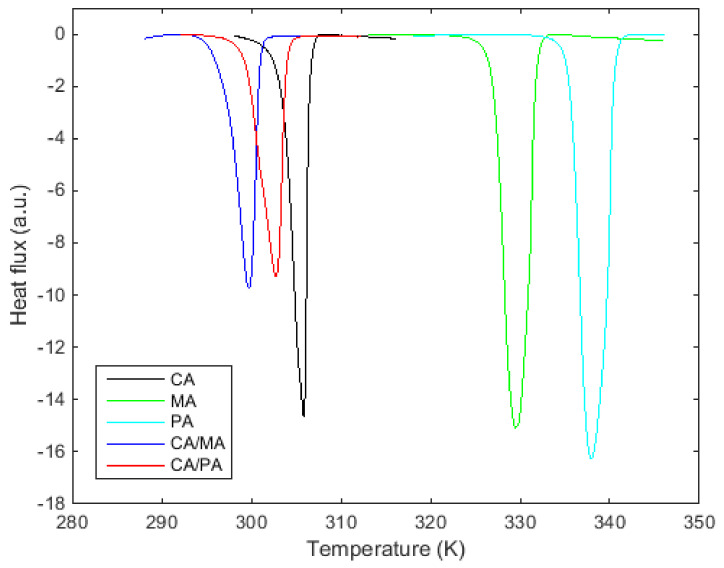
DSC curves of the three pure fatty acids and the two eutectic compositions.

Figure 1 shows that the estimated eutectic compositions of CA/MA and CA/PA listed in Table 3 behave as the tested pure fatty acids showing only one peak (one transition) in the DSC curve.

### 3.2. Densities

**Figure 2:**
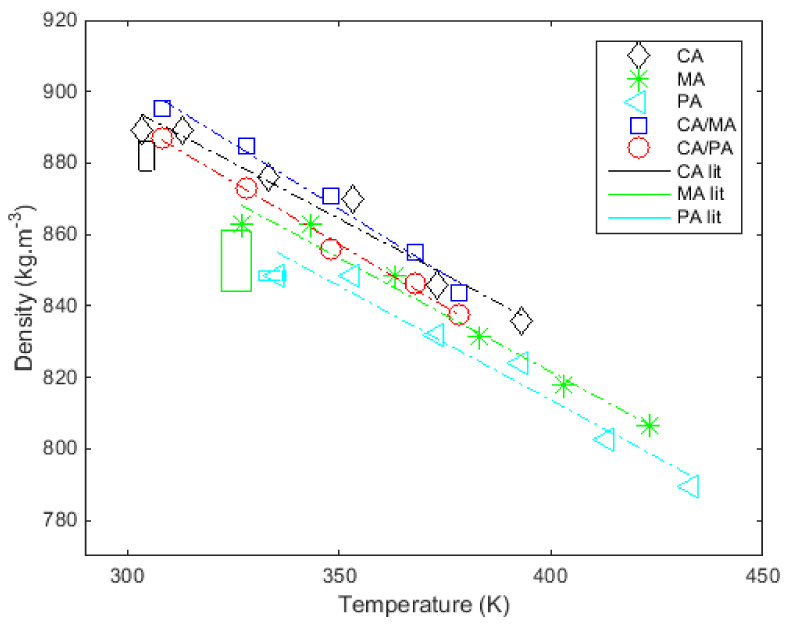
Measurements of the density in the liquid state as a function of temperature and comparison with results from the literature at melting temperature ([28,38,44,45,52,59,60]).

Figure 2 shows the densities of fatty acids and their mixtures in liquid state according to temperature (see the last column in Table 5). Experimental data are represented with symbols and linear regressions obtained (whose coefficients are adjusted to minimize the difference with the experimental data) with continuous lines.

The densities measured in the liquid state range from 848.4 to 889 kg·m^−3^ for the tested pure fatty acids. The densities measured in the liquid state range from 887.0 to 895.3 kg·m^−3^ for the eutectic binary systems. Figure 2 shows that the liquid state of all fatty acids and mixtures decreases linearly with temperature. The temperature ranges used to determine the densities of the liquid states are shown in Table 5.

Table 5 also shows the parameters a and b of the linear regression fitted to the measured points. The adequacy of the linear regressions was confirmed by high values of the regression coefficient ($r^{2}$), which lies between 0.968 and 0.993, and low values of standard deviation $\sigma_{e}$ between 1.73 and 4.54 kg·m^−3^ (see Table 5).

The density of the pure fatty acids in the solid state at ambient temperature ranges from 989.6 kg·m^−3^ to 1016.1 kg·m^−3^ depending on the material (see Table 6). The density of the eutectic binary systems is slightly lower and ranges from 942.8 kg·m^−3^ to 989.2 kg·m^−3^ (Table 6). At ambient temperature, the densities measured for the tested pure materials in the solid state are consistent with those listed by Kenisarin [10].

As with the melting temperature and enthalpy, the density in the solid state illustrates a level of compactness or “cohesion” of the crystal network. The studied eutectic mixtures are rich in CA and relatively poor in MA and PA (<20 mol%). The compound in minority seeks to fit within the crystal network of the main compound (here CA). It is possible that it induces an increase of the average distance between molecules of CA, hence lowering the cohesive energy. It is an example of what is being described in [61]; the more extended MA and PA chains are “disturbing” the organization of CA crystals, the less cohesive they become. Consequently, the higher the cohesion energy, the higher the density. Our results are consistent in that regard and show that eutectic mixtures, having chains of different lengths, are less cohesive than the pure fatty acids composing each mixture.

Besides, at ambient temperature, the density of liquid state is always lower than the density of the solid state for all the tested materials.

Table 6 summarizes the measurements of the densities in solid $\rho_{S}$ and liquid $\rho_{L}$ states for the pure materials and the eutectic mixtures as well as an estimation of the volume expansion (Δρ), which occurs during the phase change. As the densities in the solid state were measured only at ambient temperature ($T_{amb}$), the volume expansion was estimated by the mean of the following equation knowing that this induces an approximation:

(1) $$\Delta \rho \left(T_{m}\right)=\frac{\left(\rho_{L}\left(T_{m}\right)-\rho_{S}\left(T_{amb}\right)\right)}{\rho_{L}\left(T_{m}\right)}$$

with $T_{m}$, melting temperature, $T_{amb}$, ambient temperature.

However, this allows the comparison of fatty acids and eutectic mixtures of fatty acids (the same approximation being applied to all the materials studied). The volume expansion is a significant parameter that must be taken into account when integrating the PCM into the considered thermal energy storage system. Indeed, an excessive volume expansion (>15%) could damage the PCM container and, therefore, the system. Most of the pure fatty acids exhibit volume expansions superior to 15% (see the 5th column of Table 6). Considering only the volume expansion and the densities as a selection criterion, the CA/MA would be more interesting than the CA/PA because it presents density values closer to pure fatty acids with a lower volume expansion over the phase change.

### 3.3. Specific Heat

Regarding the solid state, the specific heat of the pure materials varies from 2.08 to 2.36 J·g^−1^·K^−1^ over the tested temperature range (see last column in Table 7). The specific heat of eutectic mixtures is of the same order of magnitude (2.09 to 2.12 J·g^−1^·K^−1^).

**Figure 3:**
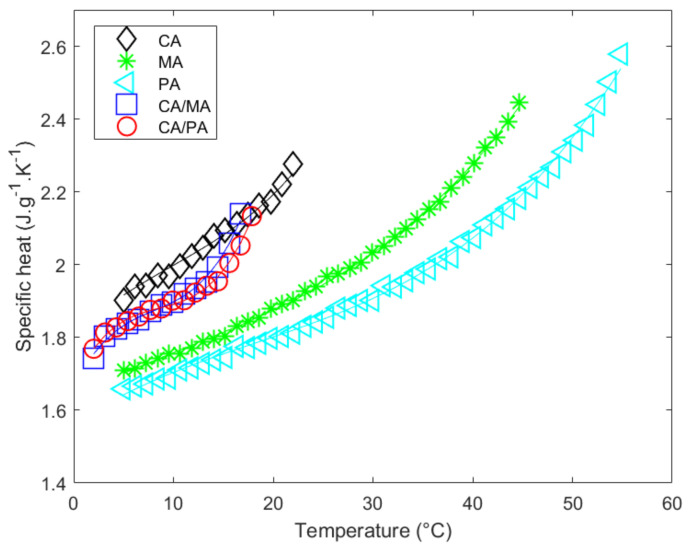
Specific heat measurements versus temperature of pure fatty acids and eutectic mixtures in the solid state.

Figure 3 depicts the measured specific heat in solid state ($C_{P,S}$) as a function of the temperature. Its evolution as a function of temperature (T) is modeled using the polynomial $C_{P,S}\left(T\right)=aT^{3}+bT^{2}+cT+d$, whose coefficients are adjusted, minimizing the difference with the measured values. The fitted coefficients a, b, c and d of this polynomial are reported in Table 7. The fitted specific heat capacity in the solid state as a temperature function is represented by continuous lines, the measured values by the symbols (Figure 3). The coherence between the data and the polynomial fit can be observed in Figure 3 and evaluated with the values of the coefficient of determination ($r^{2}$) and the standard deviation of the adjustment error ($\sigma_{e}$. See Table 7). It should be mentioned that the uncertainty of the coefficients is rather high, especially in the case of CA. It can be observed that the experimental dataset for CA appears to be quasi-linear. The form of the polynomial is therefore overcorrecting and induces a large tolerance for the determination of the coefficients. If we were to model the experimental data with a linear fit ($C_{P,S}\left(T\right)=aT+b$), the $r^{2}$ and $\sigma_{e}$ remain similar (0.9811 and 0.0156 respectively) and the coefficients and uncertainties become a=0.0202±0.0016 (J·g^−1^·K^−2^) and b=−3.7128±0.4598 (J·g^−1^·K^−1^) which shows a more reliable approach in this temperature range. For the sake of consistency and reproducibility it was chosen to use the same third order polynomial for all materials despite their showing of higher uncertainties for the determination of coefficients due to the overcorrection of the fit.

The specific heat in the liquid phase ($C_{P,L}$), is measured just above the melting temperature (see Table 8). The results obtained for the pure materials are consistent with those extracted from the literature (see Table 8).

### 3.4. Thermal Conductivities and Diffusivities

Table 9 shows the measurements of the thermal conductivities and diffusivities of the studied materials in solid state at ambient temperature obtained using the hot disk method. The experimental results are consistent with those extracted from the literature (see Table 9).

High specific heats, thermal conductivities and diffusivities allow the maximization of heat transfers and benefit from sensible heat effects. They are, therefore, of great interest to our applications. The thermal conductivities of pure fatty acids and eutectic fatty acid mixtures (0.22–0.27 W·m^−1^·K^−1^) are similar to those of paraffin waxes [32]. The thermal diffusivity of eutectic mixtures (1.480–1.151 × 10^−7^ m^2^·s^−1^) is of the same order of magnitude as the pure fatty acid ones (1.355–2.064 × 10^−7^ m^2^·s^−1^).

### 3.5. Viscosity Measurement

The results obtained for all fatty acids and their mixtures show that the viscosity curve as a function of the shear rate, at a constant temperature, is a horizontal line. Therefore, the Newtonian behavior of these liquid states is verified at the low shear rates applied (40 s^−1^–300 s^−1^). Considering these behaviors, their viscosity was measured according to temperature and at a constant shear rate (100 s^−1^). The viscosities and temperatures at which they were measured are reported in Table 10.

**Figure 4:**
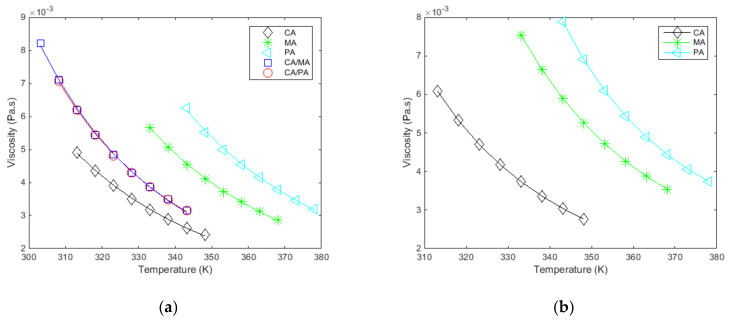
(**a**). Measured and fitted viscosities (η) as a function of the temperature (T), at a constant shear rate (100 s^−1^), for the studied materials. (**b**). Results from [62] for the 3 pure fatty acids.

**Figure 5:**
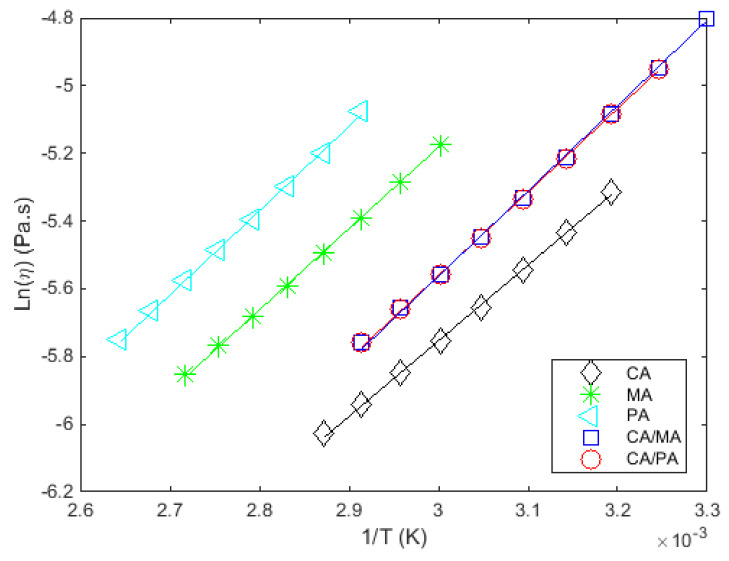
$\ln\left(\eta \right)$ vs. (1/T) at a constant shear rate (100 s^−1^) for the studied materials.

The measured and fitted viscosities as a function of temperature, at a constant shear rate, for all the studied materials are given in Figure 4a and Figure 5. Figure 4b shows the results extracted from Noureddini et al. [62] to illustrate the consistency between the results.

The viscosity progressively increases as the temperature decreases with relatively low variations (all ranging from 2 to 9 mPa·s). The measured viscosities are low compared to paraffin waxes (about 50 mPa·s at temperatures close to melting [51]), which allow a faster discharge from a thermal energy storage system which integrates fatty acids.

According to the literature, the temperature–viscosity model commonly used is the Arrhenius model (Equation (2)):

(2) $$\eta \left(T\right)=\eta_{\infty }\exp\left(\frac{E_{a}}{RT}\right)$$

where $\eta_{\infty }$ is the viscosity at infinite temperature, R is the universal gas constant, and $E_{a}$ the energy of activation.

The adequate depiction of viscosity for these materials in this temperature range by the selected model in Figure 5 is confirmed given the high values of the coefficients of determination ($r^{2}$) and the low standard deviation of the adjustment error ($\sigma_{e}$) in Table 11. It is noteworthy that the viscous flow activation energy for all fatty acids and their mixtures is comparable, highlighting similar trends in the variation of viscosity with temperature.

### 3.6. Energy Density

The energy density of the pure fatty acids and of the two eutectic compositions are calculated as follows (Equation (3)):

(3) $$E=\frac{\Delta H_{m}\times \rho_{L}\left(T_{m}\right)}{3600}$$

with E the energy density in kWh·m^−3^, $\Delta H_{m}$ the enthalpy of fusion in J·g^−1^ and $\rho_{L}$ the density in the liquid state in kg·m^−3^.

The energy densities calculated for the studied materials are listed in Table 12. The energy densities obtained (ranging from 34.15 kWh·m^−3^ to 49.93 kWh·m^−3^) are encouraging because they are similar to those of paraffins commonly used in storage systems. As the eutectic mixtures present energy densities close to those of paraffins, these materials thus constitute a promising alternative to paraffins with their advantageous thermophysical properties, their low cost and their renewable origin.

## 4. Discussion

Three pure fatty acids (capric, myristic, and palmitic acids) and two fatty acid-based eutectic mixtures (capric/myristic acids, capric/palmitic acids) have been fully characterized for LHTES applications. This study is focused on the principal thermophysical properties of PCM, such as melting temperature and enthalpy, specific heat, thermal conductivity, diffusivity, heat capacity, density and viscosity according to the temperature.

The main properties of the tested fatty acids and mixtures can be outlined as follows:

- melting temperatures ranging from 297 K (24 °C) to 335 K (62 °C)
- melting enthalpies similar to those of paraffin, with values ranging from 137 J·g^−1^ to 212 J·g^−1^
- density both in solid state (942–1016 kg·m^−3^) and in liquid state (848–896 kg·m^−3^), slightly lower than those of sugar alcohols and hydrated salts but higher than those of paraffins
- specific heats ranging from 1.66 to 2.54 J·g^−1^·K^−1^ in solid state, and from 2.12 to 2.40 J·g^−1^·K^−1^ in liquid state, values comparable to those of paraffin waxes
- relatively low thermal conductivities (~0.3 W·m^−1^·K^−1^) but slightly higher than those of paraffin
- low values of thermal diffusivities and heat capacities, as with paraffin waxes and other organic PCM
- very low viscosities (ranging from 2 to 9 mPa·s) compared to those of paraffin waxes
- energy densities (ranging from 34.15 kWh·m^−3^ to 49.93 kWh·m^−3^) similar to those of paraffin waxes commonly used in storage systems.

A comparison is made in Table 13 based on key thermophysical properties between the studied eutectic mixtures (CA/MA and CA/PA), an inorganic salt PCM (CaCl_2_-6H_2_O) in the temperature range of our study and a commercial paraffin wax (PureTemp 25) typically used for this application.

The CaCl_2_-6H_2_O hydrated salt chosen as example shows, unsurprisingly, better thermophysical properties than the organic PCM, such as the eutectic mixtures of fatty acids and the PureTemp 25 paraffin. Although their interesting thermal properties make them attractive PCM candidates, their lack of stability and hazardousness implies numerous and costly considerations to be effectively implemented in LHTES systems. The commercial paraffin and the eutectic mixtures of fatty acids appear to have very similar properties, which is also valid compared to other commercial paraffin-based PCM [32,33]. The densities of fatty acids and their eutectic mixtures in both solid and liquid states are slightly lower than those of sugar alcohols and hydrated salts [12] but are higher than those of paraffin waxes (e.g., density of Rubitherm paraffin waxes RT21, RT 25 and RT 28 are inferior to 800 kg·m^−3^ [32,33]) which is in favor of the replacement of these paraffin waxes by fatty acids in LHTES systems. The data regarding the cost of organic PCM-based TES systems is still scarce and difficult to assess. However, fatty acids are thought to be relatively inexpensive on a large scale [22]. Given the range of their thermophysical properties, they seem to be up-and-coming candidates to replace paraffin waxes in TES systems.

## 5. Conclusions

The fatty acids-based eutectic mixture of capric and myristic acids constitute a promising biobased alternative to paraffin waxes for latent heat storage at low temperatures to maintain a comfortable temperature in a building (heating/cooling depending on the time of day and the outside temperature). Indeed, it shows advantageous thermophysical properties, a low cost, a renewable origin and a low hazard (neither toxic, corrosive, explosive, nor flammable). This complete characterization of fatty acid-mixtures highlights their potential as eco-friendly PCM.

Although some of the tested fatty acids and their mixtures, particularly the eutectic mixture capric/myristic acid, seem very promising for short-term thermal energy storage for building applications, many tests are still required for their application in a real environment. For instance, studies of long-term thermal and chemical stability must be carried out to verify if the selected materials can endure a large number of fusion-solidification (i.e., charge-discharge) cycles without alteration of their properties and performances. This study must be multi-scale (material integrated into the reactor). Besides, a systematic and 3D study of crystal growth should be considered to know whether the reachable powers are sufficient for the intended application. Further works will focus on their integration in low-temperature latent heat storage systems.

## Figures and Tables

**Table 1:** Comparison of paraffins and fatty acids properties [29,34,35,36].

Selection Criteria	Paraffins RT21, 25, 26, PureTemp 25	Selected Fatty Acids
Toxicity	yes	no
Flammability	no	no
Chemical Reactivity	no	no
Biobased PCM	no	yes
Purity *	–	≥98%
Melting temperature (°C)	from 19 to 30	from 24 to 62
Undercooling	yes	low
Enthalpy (J·g^−1^)	from 150 to 230	from 137 to 212
Energy storage density (kWh·m^−3^)	35–55	35–54
Price (€/kg)	~5	~3

* Purity data is not provided by the suppliers of commercial paraffins.

**Table 2:** General information about the studied fatty acids.

Fatty Acid	Acronym	CAS Number	Formula	Supplier	Purity * (%)
Capric acid (Decanoic acid)	CA	334-48-5	C10H20O2	Alfa Aesar	99
Myristic acid (Tetradecanoic acid)	MA	544-63-8	C14H28O2	Acros Organics	99
Palmitic acid (Hexadecanoic acid)	PA	57-10-3	C16H32O2	Acros Organics	99

* Purity as given by the supplier.

**Table 3:** Fatty acids eutectic mixtures [22].

Binary System	Acronym	Molar Composition (%)	Solidification Temperature (°C)	Melting Temperature (°C)
Capric acid–myristic acid	CA/MA	83–17	21.36	24.14
Capric acid–palmitic acid	CA/PA	88–12	24.58	26.10

**Table 4:** Melting temperatures and enthalpies obtained using the DSC (this work) compared to those from the literature [25,26,28,38,40,44,45,46,47,48,49,50,51,52,53,54,55,56,57,58] at pressure *p* = 0.1 MPa *.

Materials		This Work		Previous Works	
	$T_{m}$ (°C) DSC	$T_{m}$ (K) DSC	$\Delta H_{m}$ (J·g^−1^) DSC	$T_{m}$ (K) Literature	$\Delta H_{m}$ (J·g^−1^) Literature
CA	30.41	303.41	167.97	302.77–306.30 [25,26,28,40,44,46,47,48,49,50,51,53,57,58]	139.77–168.77 [25,26,28,40,44,46,47,48,49,50,51,53,57,58]
MA	53.98	326.98	195.44	322.15–329.21 [25,26,28,38,44,45,47,48,49,50,51,52,53,54,55,56,57,58]	178.14–210.70 [25,26,28,38,44,45,47,48,49,50,51,52,53,54,55,56,57,58]
PA	62.35	335.35	211.85	331.24–337.15 [25,26,28,38,40,44,47,48,49,50,51,52,53,55,56,57,58]	185.40–233.24 [25,26,28,38,40,44,47,48,49,50,51,52,53,55,56,57,58]
CA/MA	24.29	297.29	137.3	293.23–299.17 [26,48,49,51,58]	147.70–155.20 [26,48,49,51,58]
CA/PA	26.25	299.25	148.14	295.25–301.86 [26,40,48,49,51,58]	141.40–153.00 [26,40,48,49,51,58]

* Standard uncertainties given for a 95% confidence interval for our measurements are u($T_{m}$) = 0.5 °C, u($\Delta H_{m}$) = 5% and u(p) = 10 kPa.

**Table 5:** Density in liquid state—Estimated values of the coefficients a and b in the equation $\rho \left(T\right)=aT+b$ ($r^{2}$ = regression coefficient; $\sigma_{e}$ = standard deviation of the error; T = temperature) at pressure *p* = 0.1 MPa *.

Materials	a (kg·m^−3^·K^−1^)	b (kg·m^−3^)	$r^{2}$	$\sigma_{e}$ (kg·m^−3^)	Temperature Range (K)
CA	−0.6835 ± 0.2287	918.06 ± 19.403	0.9679	4.5444	[313; 393]
MA	−0.7148 ± 0.0873	912.02 ± 9.910	0.9956	1.7340	[343; 423]
PA	−0.7349 ± 0.1614	907.54 ± 19.894	0.9859	3.2068	[353; 433]
CA/MA	−0.7313 ± 0.1562	923.33 ± 12.083	0.9867	2.8107	[308; 378]
CA/PA	−0.6981 ± 0.1104	910.91 ± 8.5375	0.9927	1.9860	[308; 378]

* Standard uncertainty given for a 95% confidence interval for our measurements is u(p) = 10 kPa. The uncertainty given for the fitting parameter is given for a 95% confidence interval.

**Table 6:** Densities at ambient temperature in the solid state ($\rho_{S}$), at melting temperature in the liquid state ($\rho_{L}$) and volume expansion (Δρ) of the three pure fatty acids and the two eutectic compositions. Comparison with the literature ([28,38,44,45,52,59,60]) at pressure *p* = 0.1 MPa *.

Materials	This Work				Previous Works	
	$T_{m}$ (K)	$\rho_{S}$$\;(T_{amb})$ (kg·m^−3^)	$\rho_{L}$$\;(T_{m})$ (kg·m^−3^)	Δρ (%)	$\rho_{S}$$\;(T_{amb})$ (kg·m^−3^)	$\rho_{L}$$\;(T_{m})$ (kg·m^−3^)
CA	303.41	1016.1 ± 3.7	889.0 ± 19.8	14.3 ± 2.6	850–1004 [28,44,59]	878–886 [28,44,59]
MA	326.98	1000.2 ± 0.9	862.8 ± 18.6	15.9 ± 2.5	860–990 [28,38,44,45,52,59,60]	844–861 [28,38,44,45,52,59,60]
PA	335.35	989.6 ± 1.7	848.4 ± 18.0	16.6 ± 2.5	900–989 [28,38,44,52,59]	847–850 [28,38,44,52,59]
CA/MA	297.29	942.8 ± 4.4	895.3 ± 20.0	5.3 ± 2.4	–	–
CA/PA	299.25	989.2 ± 1.6	887 ± 19.7	11.5 ± 2.5	–	–

* Standard uncertainties given for a 95% confidence interval for our measurements are u($T_{m}$) = 0.5 K and u(p) = 10 kPa. Uncertainties for the density in the solid state are obtained through repeated measurements and through error propagation in the liquid state.

**Table 7:** Specific heat of the solid phase—Estimated values of the coefficients a, b, and c in the equation $C_{P,S}\left(T\right)=aT^{3}+bT^{2}+cT+d$ ($r^{2}$ = coefficient of determination; $\sigma_{e}$ = standard deviation of the error) at pressure *p* = 0.1 MPa *.

Materials	a × 10^5^ (J·g^−1^·K^−4^)	b (J·g^−1^·K^−3^)	c (J·g^−1^·K^−2^)	d (J·g^−1^·K^−1^)	$r^{2}$	$\sigma_{e}$ (J·g^−1^·K^−1^)	Temperature Range (K)
CA	3.6535 ± 6.9456	−0.0310 ± 0.0597	8.7941 ± 17.1184	−830.51 ± 1635.1	0.9871	0.0140	[278; 296]
MA	0.7738 ± 0.1805	−0.0066 ± 0.0016	1.8873 ± 0.4805	−179.18 ± 47.657	0.9991	0.0068	[278; 318]
PA	0.8506 ± 0.1354	−0.0074 ± 0.0012	2.1770 ± 0.3727	−211.51 ± 37.566	0.9981	0.0112	[278; 328]
CA/MA	22.996 ± 8.9840	−0.1940 ± 0.0761	54.563 ± 21.509	−5114.5 ± 2025.0	0.9915	0.0110	[278; 291]
CA/PA	22.856 ± 4.8510	−0.1932 ± 0.0412	54.445 ± 11.661	−5113.0 ± 1100.0	0.9949	0.0077	[278; 290]

* Standard uncertainty given for a 95% confidence interval for our measurements is u(p) = 10 kPa. The uncertainty given for the fitting parameter is given for a 95% confidence interval.

**Table 8:** Comparison of the specific heat capacities measured in the solid and the liquid states close to the melting temperature in this study and extracted from the literature [59,60] at pressure *p* = 0.1 MPa *.

Materials	This Work			Previous Works	
	$T_{m}$ (K)	$C_{P,S}$ (J·g^−1^·K^−1^)	$C_{P,L}$$\left(T_{m}\right)$ (J·g^−1^·K^−1^)	$C_{P,S}$ (J·g^−1^·K^−1^)	$C_{P,L}$$\left(T_{m}\right)$ (J·g^−1^·K^−1^)
CA	303.41	1.88–2.30 ± 0.05	2.14 ± 0.04	2.09–2.10 [59]	1.90–3.00 [59]
MA	326.98	1.72–2.46 ± 0.03	2.36 ± 0.02	2.16–2.40 [59,60]	1.70–2.18 [59,60]
PA	335.35	1.66–2.54 ± 0.02	2.39 ± 0.02	2.27–2.8 [59]	1.90–2.06 [59]
CA/MA	297.29	1.83–2.05 ± 0.08	2.40 ± 0.05	–	–
CA/PA	299.25	1.85–2.05 ± 0.07	2.12 ± 0.05	–	–

* Standard uncertainties given for a 95% confidence interval for our measurements are u($T_{m}$) = 0.5 K and u(p) = 10 kPa. Uncertainties for the specific heat are calculated from error propagation.

**Table 9:** Measured thermal conductivity and diffusivity in solid state at ambient temperature of fatty acids and eutectic mixtures compared with the literature [59,60] at pressure *p* = 0.1 MPa *.

Materials	This Work		Previous Work	
	k (W·m^−1^·K^−1^)	α × 10^7^ (m^2^·s^−1^)	k (W·m^−1^·K^−1^)	α (m^2^·s^−1^) × 10^7^
CA	0.2195 ± 0.0067	1.364 ± 0.043	0.21 [59]	1.2 [59]
MA	0.2524 ± 0.0031	2.064 ± 0.146	0.17–0.39 [59,60]	2 [59,60]
PA	0.2526 ± 0.0106	1.870 ± 0.053	0.3 [59]	1.7 [59]
CA/MA	0.2717 ± 0.0103	1.480 ± 0.072	–	–
CA/PA	0.2358 ± 0.1151	1.151 ± 0.133	–	–

* Standard uncertainty given for a 95% confidence interval for our measurements is u(p) = 10 kPa. Uncertainties for the thermal conductivity and diffusivity is obtained from repeated measurements.

**Table 10:** Measured viscosities of the materials studied at pressure *p* = 0.1 MPa *.

CA		MA		PA		CA/MA		CA/PA	
T (°C)	η (mPa·s)	T (°C)	η (mPa·s)	T (°C)	η (mPa·s)	T (°C)	η (mPa·s)	T (°C)	η (mPa·s)
40	4.91 ± 0.04	60	5.67 ± 0.02	70	6.25 ± 0.01	30	8.21 ± 0.02	35	7.08 ± 0.01
45	4.36 ± 0.01	65	5.06 ± 0.01	75	5.53 ± 0.12	35	7.11 ± 0.03	40	6.19 ± 0.01
50	3.90 ± 0.02	70	4.55 ± 0.02	80	5.00 ± 0.04	40	6.19 ± 0.02	45	5.44 ± 0.02
55	3.50 ± 0.02	75	4.11 ± 0.02	85	4.54 ± 0.04	45	5.45 ± 0.03	50	4.81 ± 0.03
60	3.17 ± 0.02	80	3.73 ± 0.01	90	4.15 ± 0.02	50	4.84 ± 0.02	55	4.30 ± 0.03
65	2.88 ± 0.02	85	3.41 ± 0.01	95	3.79 ± 0.01	55	4.30 ± 0.03	60	3.86 ± 0.02
70	2.63 ± 0.02	90	3.13 ± 0.02	100	3.47 ± 0.02	60	3.87 ± 0.02	65	3.49 ± 0.02
75	2.41 ± 0.02	95	2.87 ± 0.02	105	3.19 ± 0.02	65	3.49 ± 0.03	70	3.16 ± 0.03
–	–	–	–	–	–	70	3.16 ± 0.03	–	–

* Standard uncertainties given for a 95% confidence interval for our measurements are u(T) = 1 °C and u(p) = 10 kPa. Uncertainties for the viscosities are obtained from repeated measurements.

**Table 11:** Viscosity—Estimated values of the coefficients $\eta_{\infty }$ and $E_{a}/R$ of the equation $\eta \left(T\right)=\eta_{\infty }\exp\left(\frac{E_{a}}{RT}\right)$ ($r^{2}$ = regression coefficient; $\sigma_{e}$ = standard deviation of the error) at pressure *p* = 0.1 MPa *.

Materials	$\eta_{\infty }$ × 10^−6^ (Pa·s)	$E_{a}/R$ (K)	$r^{2}$	$\sigma_{e}$ × 10^9^ (Pa·s)	Temperature Range (K)
CA	3.8674 ± 0.6950	2236.5 ± 57.6	0.99955	0.32376	[313; 348]
MA	4.1726 ± 0.7215	2401.7 ± 59.7	0.99941	0.56511	[333; 368]
PA	4.3810 ± 1.1159	2488.8 ± 90.5	0.9991	1.4171	[343; 378]
CA/MA	2.0070 ± 0.4128	2518.8 ± 65.7	0.99919	2.3031	[303; 343]
CA/PA	2.3638 ± 0.4695	2464.9 ± 64.6	0.99931	1.2000	[308; 343]

* Standard uncertainty given for a 95% confidence interval for our measurements is u(p) = 10 kPa. The uncertainty given for the fitting parameter is given for a 95% confidence interval.

**Table 12:** Energy densities calculated for the studied materials at pressure *p* = 0.1 MPa *.

Materials	E (kWh·m^−3^)
CA	41.48 ± 2.27
MA	46.84 ± 2.55
PA	49.93 ± 2.71
CA/MA	34.15 ± 1.87
CA/PA	36.50 ± 2.00

* Standard uncertainty given for a 95% confidence interval for our measurements is u(p) = 10 kPa. Uncertainties for the energy density is calculated from error propagation.

**Table 13:** Comparison of PCM in the temperature range of application.

Materials	$T_{m}$ (K)	$\Delta H_{m}$ (J·g^−1^)	ρ (kg·m^−3^)		$C_{P}$ (J·g^−1^·K^−1^)		k (W·m^−1^·K^−1^)	E (kWh·m^−3^)
			Sol. (T_amb_)	Liq. (T_m_)	Sol.	Liq.	Sol.	
CA/MA	297.29	137.3	942.8	895.3	1.83–2.05	2.40	0.2717	34.15
CA/PA	299.25	148.14	989.2	887	1.85–2.05	2.12	0.2358	36.50
CaCl_2_-6H_2_O [44]	302.75	191	1802	1562	1.42	2.10	1.088	82.87
PureTemp 25 [33]	298.15	187	950	860	1.99	2.29	–	44.67

## Data Availability

The data presented in this study are available on request from the corresponding author.
